# Severe Pulmonary Hypertension and Cholestatic Liver Injury: Two Rare Manifestations of Graves’ Disease

**DOI:** 10.7759/cureus.9236

**Published:** 2020-07-17

**Authors:** Bisrat Nigussie, Fuad I Abaleka, Tigist Gemechu, Maham Suhail, Sukaina Alikhan

**Affiliations:** 1 Internal Medicine, Richmond University Medical Center, Staten Island, USA; 2 Medicine, Richmond University Medical Center, Staten Island, USA

**Keywords:** pulmonary hypertension, cholestatic liver injury, hyperthyroidism, graves' disease, hyperbilirubinemia

## Abstract

Graves’ disease is seen in the majority of patients diagnosed with hyperthyroidism. The usual symptoms of Graves’ disease are tremors, weight loss, sweating, exophthalmos, pretibial edema, anxiety, and palpitations. However, there could be unusual manifestations such as hepatic failure and pulmonary hypertension. We present the case of a 31-year-old female with Graves’ disease with these two rare complications, which resolved with medical management in about three months. To the author’s knowledge, this is the second case of a patient with this type of presentation.

## Introduction

The mechanism for cholestatic liver injury and severe pulmonary hypertension as a complication is not well understood. Hepatic dysfunction in patients with thyrotoxicosis may be caused by hyperthyroidism as a side effect of drugs used to treat hyperthyroidism, autoimmune hepatitis, viral hepatitis, alcoholic hepatitis, and sepsis [1]. In addition, there are several causes of pulmonary hypertension, though the majority of which are classified under idiopathic. Some of the common causes of pulmonary hypertension are chronic pulmonary embolism, left-sided heart failure, chronic obstructive pulmonary disease (COPD), sleep apnea, connective tissue disease such as systemic sclerosis, and Graves’ disease [2]. In Graves’ disease, mild pulmonary hypertension is common, but severe cases are rare [3].

## Case presentation

A 31-year-old African American female presented to the hospital with palpitations, shortness of breath, and weakness. She had a known history of Graves’ disease but was non-compliant with medications. She complained of palpitations over the last several weeks and noticed her eyes were turning yellow during this time frame. On examination, the patient did not appear to be in any distress, blood pressure was 151/92 mmHg, heart rate was 122 bpm, and respiratory rate was 22 breath per minute. She had icteric sclera, exophthalmos, and lid lag. Also, she had an enlarged goiter that measured 4 x 5 cm in size. The goiter was shaped like a butterfly, it was symmetrical, it involved bilateral anterior triangles of the neck, it was firm to palpation, it moved with deglutition, its borders were well defined, and it was non-tender. Furthermore, she had distended neck veins but no lymphadenopathy. Abdominal examination revealed a distended abdomen with positive fluid shift and palpable liver 3 cm below the right costal margin along with the midclavicular line. Furthermore, she had 3+ bilateral lower extremity edema and fine resting tremors.

Laboratory investigation revealed total bilirubin of 13 mg/dL, direct bilirubin of 8.6 mg/dL, alkaline phosphatase (ALP) of 167 mg/dL, aspartate aminotransferase (AST) of 55 mg/dL, and alanine aminotransferase (ALT) of 22 mg/dL. International normalized ratio (INR) was mildly elevated at 1.5. Thyroid-stimulating hormone was 0.0006 mIU/mL, free T4 was 7.44 ng/dL, and free T3 was 6.25 nmol/L. See Table 1 for further lab values. Antinuclear antibody, antimitochondrial antibody, antineutrophil cytoplasmic antibody, hepatitis B surface antigen, hepatitis B surface antibody, hepatitis C antibody, and human immunodeficiency virus (HIV) were all negative. Ceruloplasmin and alpha-1 antitrypsin were slightly elevated.

**Table 1:** Lab thyroid and hepatic function test results. TSH, thyroid-stimulating hormone; ALP, alkaline phosphatase; AST, aspartate aminotransferase; ALT, alanine aminotransferase

	At admission	1 week	2 weeks	10 weeks
Bilirubin total (mg/dL)	13	8.71	4.58	2.47
Direct (mg/dL)	8.7	5.5	2.8	1.3
Indirect (mg/dL)	4.6	3.2	1.6	1.1
TSH (mIU/ml)	0.0006		0.0003	1.0
Free T4 (ng/dL)	7.44			2.1
Free T3 (nmol/L)	6.25			3.2
ALP (mg/dL)	167	127	115	96
AST (mg/dL)	55	48	39	31
ALT (mg/dL)	22	22	21	20

**Figure 1:**
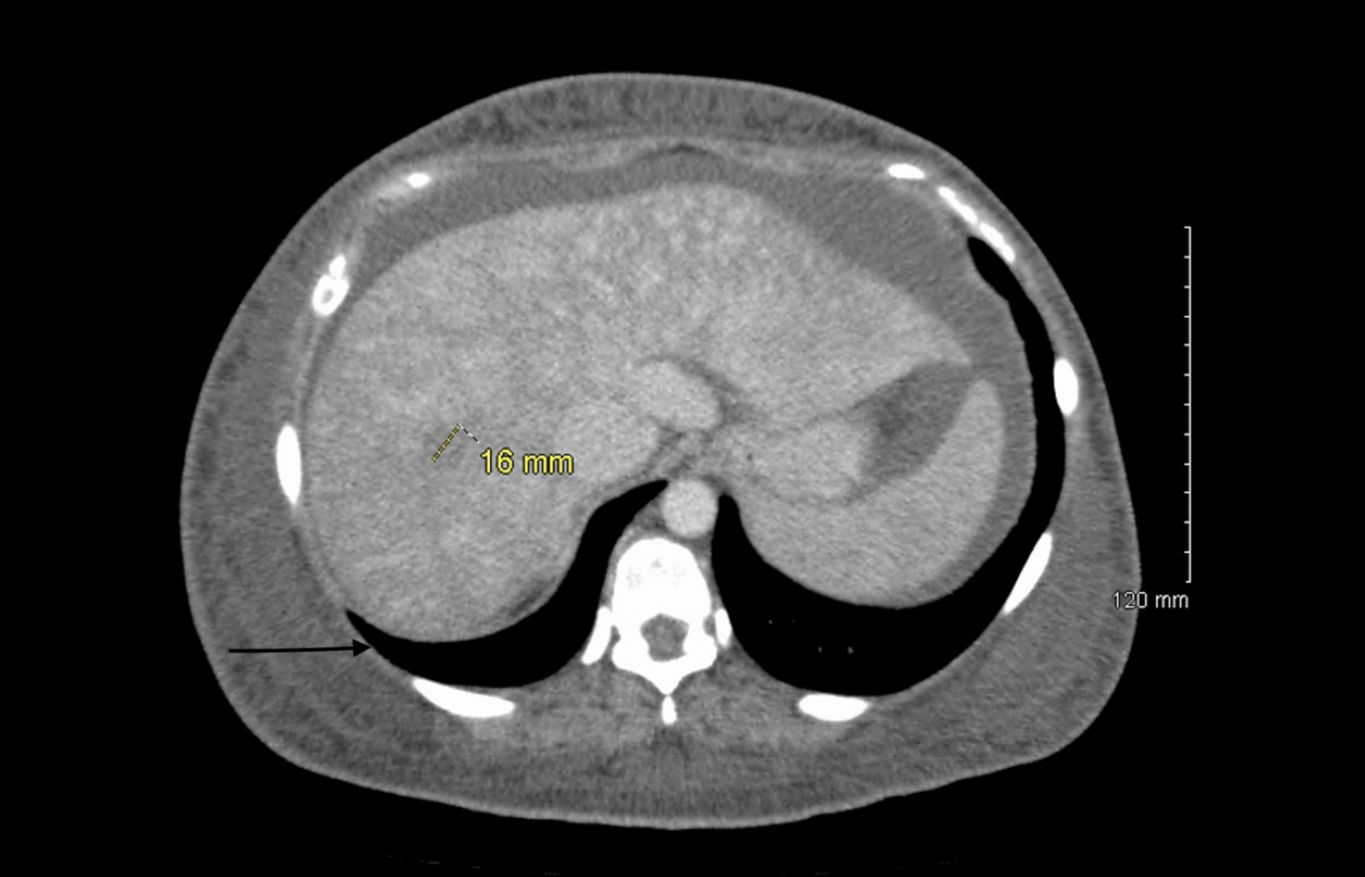
CT scan with the black arrow showing mild abdominal ascites and a 16-mm mass at the junction of segments VII and VIII of the liver, which is suggestive of congestive hepatomegaly.

Abdominal ultrasound showed mild hepatomegaly with a heterogeneous liver, distended inferior vena cava, and hepatic vein findings suspicious for congestive hepatomegaly. A CT scan was performed, which showed severe generalized anasarca and a small amount of abdominal ascites as well as a moderate amount of pelvic ascites, as shown in Figure 1. The liver appeared to be moderately heterogeneous with an ill-defined focal load.

Echocardiogram showed left ventricle ejection fraction of 60%, right ventricle systolic pressure (RVSP) of 58.18 mmHg (normal is <35 mmHg), and moderate tricuspid regurgitation. Thyroid uptake scan from previous outpatient clinic data had shown diffuse increase uptake.

The patient was diagnosed with atrial fibrillation with a rapid ventricular rate secondary to thyrotoxicosis. Also, she was found to have pulmonary hypertension with right-sided heart failure and cholestatic liver injury. She was treated with propranolol, methimazole, furosemide, dexamethasone, and iodine. In the subsequent follow-ups at 1 week, 2 weeks, and 10 weeks, she had resolution of cholestatic hepatic injury as well as improvement of right-sided heart failure.

## Discussion

Our patient had two rare manifestations of Graves’ disease, which are severe pulmonary hypertension and cholestatic liver injury. Mild pulmonary hypertension can be seen in 36-65% of Graves’ disease patients. However, it is rare to have severe pulmonary hypertension with RVSP of 58 mmHg, leading to right-sided heart failure and hepatic congestion due to Graves’ disease [3-5]. There is a clear correlation with thyroid-stimulating hormone receptor antibody and pulmonary vascular resistance and cardiac output, but the mechanism has not been established [3]. Nevertheless, some of the theories are autoimmune-mediated endothelial remodeling; mechanical endothelial damage caused by the high cardiac output; accelerated metabolism of pulmonary vasodilators such as nitric oxide and prostacyclin; inhibited metabolism of pulmonary vasoconstrictors such as endothelin-1, serotonin, and thromboxane; and enhanced pulmonary vascular response to catecholamines [6-7]. Furthermore, with the treatment of Graves’ disease, pulmonary hypertension improved. In our patient’s case, as evidenced by the transthoracic echocardiogram, pulmonary hypertension improved (RVSP decreased from 58 mmHg to 47 mmHg). The patient also had clinical evidence of improvement of pulmonary hypertension as both her shortness of breath and lower extremity edema improved. Other potential causes of pulmonary hypertension such as HIV, COPD, sleep apnea, connective tissue disorders, and obesity were ruled out.

Cholestatic hepatic injury is an uncommon complication of Graves’ disease, and the underlying mechanism is still not well understood. However, it can be seen with or without heart failure, as documented in a few case reports [8]. Some of the proposed mechanisms for cholestatic hepatic injury in hyperthyroidism are mitochondria-induced apoptosis, oxidative stress from the hyperthyroid state, and other autoimmune hepatobiliary diseases. In our patient’s case, other causes of liver injury such as autoimmune hepatobiliary diseases, infectious causes, and storage diseases (e.g. Wilson’s and hemochromatosis) were ruled out. Also, the patient was not taking any medication that is known to cause liver injury. However, the patient had congestive hepatomegaly due to right-sided heart failure. Patients with congestive hepatomegaly will have elevated AST and ALT with mild bilirubin and ALP [9]. In our patient’s case, the elevation in liver enzyme was more of a cholestatic pattern (highly elevated direct bilirubin and three times the normal ALP), which resolved following the treatment of Graves’ disease. Our patient had two rare manifestations of Graves’ disease: severe pulmonary hypertension and cholestatic liver injury.

## Conclusions

Severe pulmonary hypertension leading to right heart failure and severe cholestatic liver injury are two uncommon presentations of Graves’ disease. They are even more uncommon when presenting together in one patient. Proper diagnosis and management of Graves’ disease mitigated the worsening of hepatic and cardiac failure.
